# Algorithmic processing of pressure waveforms to facilitate estimation of cardiac elastance

**DOI:** 10.1186/1475-925X-11-28

**Published:** 2012-06-15

**Authors:** David Stevenson, James Revie, J Geoffrey Chase, Christopher E Hann, Geoffrey M Shaw, Bernard Lambermont, Alexandre Ghuysen, Philippe Kolh, Thomas Desaive

**Affiliations:** 1 Department of Mechanical Engineering, Centre for Bio Engineering at the University of Canterbury, Christchurch, New Zealand; 2 Department of Intensive Care, Christchurch Hospital, Christchurch, New Zealand; 3 Cardiovascular Research Center, University of Liege, Belgium

## Abstract

**Background:**

Cardiac elastances are highly invasive to measure directly, but are clinically useful due to the amount of information embedded in them. Information about the cardiac elastance, which can be used to estimate it, can be found in the downstream pressure waveforms of the aortic pressure (*P*_*ao*_) and the pulmonary artery (*P*_*pa*_). However these pressure waveforms are typically noisy and biased, and require processing in order to locate the specific information required for cardiac elastance estimations. This paper presents the method to algorithmically process the pressure waveforms.

**Methods:**

A shear transform is developed in order to help locate information in the pressure waveforms. This transform turns difficult to locate corners into easy to locate maximum or minimum points as well as providing error correction.

**Results:**

The method located all points on 87 out of 88 waveforms for *P*_*pa*_, to within the sampling frequency. For *P*_*ao*_, out of 616 total points, 605 were found within 1%, 5 within 5%, 4 within 10% and 2 within 20%.

**Conclusions:**

The presented method provides a robust, accurate and dysfunction-independent way to locate points on the aortic and pulmonary artery pressure waveforms, allowing the non-invasive estimation of the left and right cardiac elastance.

## Background

In an Intensive Care Unit (ICU), cardiac disturbances are difficult to diagnose and treat, which can lead to poor management [1,2]. Inadequate diagnosis can be common, and plays a significant role in increased length of stay and death [3-5], despite access to many different cardiac measurements and metrics. Currently, internal measurements are only possible at the locations where catheters are placed. This limited set of data can severely restrict clinical diagnostic capability, and thus these catheters are not necessarily associated with improved outcomes [6-8]. Overall, a lot of data currently available to ICU clinicians, that could have significant clinical value, is under utilised.

Using modelling techniques, this limited set of data can be expanded to estimate a much greater set of clinically relevant data to enable more accurate diagnosis. For example, acute cardiovascular dysfunction, like pulmonary embolism (PE) and septic shock, severely alter \ system (CVS) hemodynamics around the heart. These changes can be seen by catheter measurements as a change in the balance of preload and afterload, resulting in an altered cardiac energetic state [9,10]. Detailed cardiac energetics are too invasive to measure in an ICU setting. However, if the relevant energetics could be captured from a nearby catheter, the clinical potential of such measurements could be realised. To date, no such method achieves this aim.

Time-varying cardiac elastance (TVE) is defined [11]:

(1) $$e(t)=\frac{P_{v}(t)}{V_{v}(t)-V_{d}}$$

where, *V *_*d*_ is assumed to be equal to *V *_0_ for simplicity, *V *_0_ is the intercept of the end-systolic pressure-volume relation (ESPRV) with the volume axis [12], *P*_*v*_(*t*) is the ventricle pressure and *V *_*v*_(*t*) is the ventricle volume. It thus provides a measure of heart function and energetics [13-15]. The waveform *e*(*t*) is typically normalised to a value of 1.0 [13], and can also be used as the input or driver function in lumped parameter CVS models [16-20].

There have been several attempts to estimate TVE [14,21-24]. However, none have estimated it for its own sake. Most studies present a method using the TVE to estimate a specific parameter, most commonly end-systolic elastance (*E*_*es*_) [14,23,24] and ejection fraction [22]. However, their validation is based on these metrics, not on the resulting TVE waveform.

This research is unique in that the end goal is to produce the TVE function in its own right, validating the TVE waveform on its own accuracy for eventual use as a diagnostic tool. It is unclear to date how much specific information can be obtained from the TVE waveform, other than the highly sought-after *E*_*es*_[25] (although this cannot be found from the normalised TVE waveform). However, TVE features are highly correlated to clinical parameters [26] and contain similar information to pressure-volume (PV) loops, which are known to contain information on cardiac function [27] including cardiac work [28,29], contractility [13,30], O_2_ consumption [29,31], and all the states of filling, contraction ejection and relaxation [1]. Thus, a TVE waveform reflects cardiac state, cardiac output or blood volume, and net preload and afterload, all of which change with cardiac dysfunction. Hence, the ability to easily and non-invasively obtain TVE waveforms could enable clinically useful diagnostics and metrics.

This paper presents the first step in estimating TVE from already available measurements, namely aortic (*P*_*ao*_) and pulmonary artery (*P*_*pa*_) pressure. This research develops algorithms to process these pressure waveforms to extract specific features and points, which, in turn, allow the estimation of the end goal, the TVE waveform. The *P*_*ao*_ and *P*_*pa*_ pressure waveforms, as typically measured, are noisy and/or biased, which can significantly effect this process. Hence, a method is presented for automatically processing the pressure waveforms to robustly and accurately locate the points required by the correlations, that enable accurate, not additionally invasive cardiac elastance estimation and construction.

## Methods

### Concept

**Figure 1:**
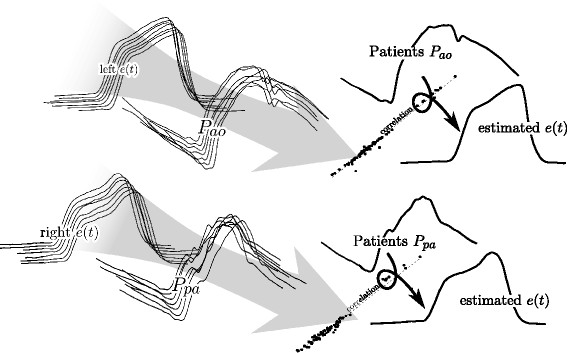
**Overview.** The figure shows a conceptualised overview of the process described in this paper and further implications. From the many measured left cardiac elastance (*e*(*t*)) waveforms, alone with many aortic pressure (*P*_*ao*_) waveforms, correlations are derived (the information flow is shown through the large grey arrow). Once these correlations are known, they can be used along with the aortic pressure waveform (from a patient), to arrive at an estimation of their cardiac elastance waveform. The equivalent for the right cardiac elastance is also shown, with the pulmonary artery pressure (*P*_*pa*_).

The specific approach presented defines a representative set of points on the TVE waveforms. These points allow a continuous waveform to be constructed that adequately follows the shape of a true (invasively measured) TVE, capturing the necessary dynamics. Correlations between these representative points and the points or properties on the typically measured waveforms, *P*_*ao*_ and *P*_*pa*_, enable construction of continuous beat-to-beat estimations of TVE with knowledge only of the *P*_*ao*_ and *P*_*pa*_ waveforms and the pre-defined correlations. It thus uses typically available data to construct what would otherwise require a highly invasive added test. A high level view of this approach is presented in Figure 1, which shows the formation and use of the correlations to generate and estimated cardiac elastance waveform. This paper is focused on the left half of Figure 1, that of producing the correlations for later use. A brief overview of the whole method is given for clarity: 

1. locate points on *P*_*ao*_ and *P*_*pa*_ (this paper)

2. correlate points of the pressure waveforms to points on the measured cardiac elastance

3. use these correlations to estimate the points on the cardiac elastance

4. create a continuous function *e*(*t*) through the estimated points with (2)-(4)

5. compare the estimated elastance waveform to the measured elastance waveform

(2) $$\begin{array}{l} e(t)=\left\{\begin{array}{ll} F_{\alpha }(t) & 0<t<c_{\alpha }\\ \frac{\left(1-x_{2})(t-c_{\alpha }\right)}{c_{\beta }-c_{\alpha }}+x_{2} & c_{\alpha }<t<c_{\beta }\\ F_{\beta }(t) & c_{\beta }<t<\text{period} \end{array}\right)\; \end{array}$$

where:

(3) $$F_{i}=a_{i}·e^{-b_{i}{\left(t-c_{i}\right)}^{2}}$$

and the coefficients of (3), also seen in (2), are fitted for a specific waveform, and are defined:

(4) $$\begin{array}{l} a_{\alpha }=x_{2}\\ b_{\alpha }=-\frac{\text{log}(x_{1}/x_{2})}{\text{exp}(\text{log}(-\text{log}(x_{1}/x_{2})·2·(x_{1}/{\dot{x}}_{1}))·2)}\\ c_{\alpha }=-\frac{\text{log}(x_{1}/x_{2})·2·x_{1}-{\dot{x}}_{1}·t_{1}}{{\dot{x}}_{1}} \end{array}$$

where *a*_*β*_, *b*_*β*_ and *c*_*β *_are similarly defined by replacing subscript 1 with 3 and setting *x*_2_ = 1.

**Figure 2:**
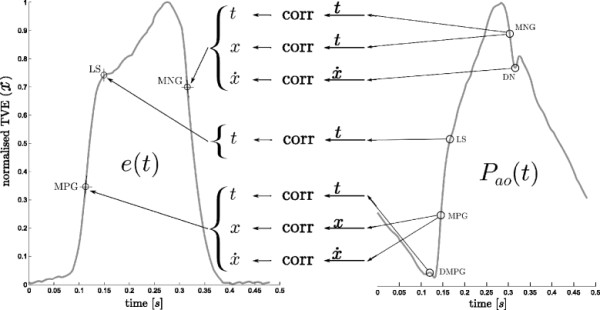
**Illustrative elastance estimation.** An example of what can be done with the identified points on the aortic pressure, and an example of the formation of the estimated cardiac elastance is shown here, while the terms are defined in (5). This figure is not part of the method of this paper, rather as a illustration of what the method as a whole leads to.

**Figure 3:**
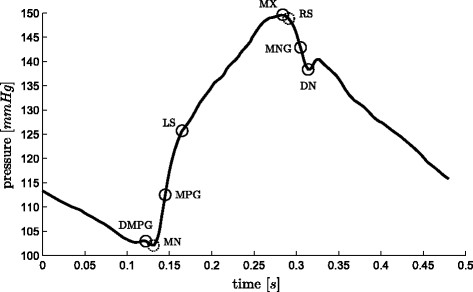
**Aortic pressure waveform and relevant points.** A representative aortic pressure waveform over one heart beat with relevant points (defined in (5)) marked on it. The two dashed circles, *MN* and *RS* are used only in locating other points.

**Figure 4:**
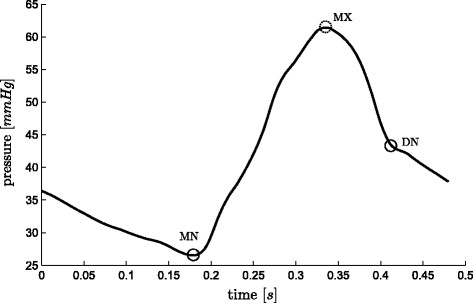
**Pulmonary artery pressure waveform and relevant points.** A representative pulmonary artery pressure waveform over one heart beat with relevant points (defined in (5)) marked on it. The dashed circle, *MX* is only used to help find other points.

Figure 2 shows an illustrative mapping between points on *P*_*ao*_ and TVE. However, this approach is useful if and only if it is possible to automate the detection of the required points, defined in (5), on the *P*_*ao*_ and *P*_*pa*_ waveforms, shown in Figures 3 and 4.

This paper focuses on the robust capturing of the points on the pressure waveforms and leaves the specific correlations and methodology of creating the TVE waveforms to a paper in review. The details contained in this paper about the formulation of the correlations and there use are illustrative only, and are assumed to be correct for the purposes of demonstrating a potential use for this research.

For describing the methods in this paper, a naming convention is defined:

(5) $$\begin{array}{rll} e(t) & \equiv & \text{time varying cardiac elastance}\\ P_{\text{ao}} & \equiv & \text{aortic pressure}\\ P_{\text{pa}} & \equiv & \text{pulmonary artery pressure}\\ \text{DMPG} & \equiv & \text{driver maximum positive gradient}\\ \text{MN} & \equiv & \text{minimum point}\\ \text{MPG} & \equiv & \text{maximum positive gradient}\\ \text{LS} & \equiv & \text{left shoulder}\\ \mathrm{MX} & \equiv & \text{maximum}\\ \text{RS} & \equiv & \text{right shoulder}\\ \text{MNG} & \equiv & \text{maximum negative gradient}\\ \mathrm{DN} & \equiv & \text{dicrotic notch} \end{array}$$

### Shear Transform

This paper uses a shear transform to extract features from the *P*_*ao*_ and *P*_*pa*_ waveforms, defined:

(6) $$S\equiv (t,X(t))\to \left(t,\phi_{\text{shear}}(X(t))\right)$$

where:

(7) $$\phi_{\text{shear}}\left(X(t)\right)=X(t)+\text{mt}+c,\;t_{0}<t<t_{\mathrm{end}}$$

(8) X(t)≡descrete, time valued data

and the parameters *t*_0_ and *t*_*end*_ are set depending on the region or period of interest. The parameters *m* and *c* are chosen such that:

(9) $$\phi_{\text{shear}}(X(t_{0}))=X(t_{0})=\phi_{\text{shear}}(X(t_{\text{end}}))$$

Equation (9) leads to:

(10) $$\begin{array}{r} X(t_{0})+m·t_{0}+c=X(t_{0})\\ X(t_{\text{end}})+m·t_{\text{end}}+c=X(t_{0}) \end{array}$$

Solving (10), for *m* and *c* yields:

(11) $$\begin{array}{l} m=\frac{X(t_{0})-X(t_{\text{end}})}{t_{\text{end}}-t_{0}}\\ \;c=-m·t_{0} \end{array}$$

**Figure 5:**
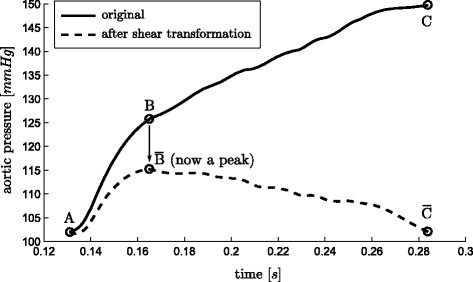
**Shear transform.** An illustration of the shear transformation of (6), turning a hard to locate “shoulder” ( $\bar{\text{B}}$) points into an easily found maximum point ( $\bar{\text{B}}$).

To better visualize how this transformation operates, imagine a line from *A* to *C* in Figure 5, representing a portion of the waveform in Figure 3, rotated about *A* so that end points align horizontally, while time remains unchanged. Hence, it is a rotation and contraction that projects the line onto a horizontal axis (time). The effect of this transformation is to transform the difficult to find “shoulder” point *B* into an easily found peak of a curve, or for the reflection of *A * →* B* in *x*, a minimum or the curve. A “shoulder” is defined as a point at which two near linear lines with different slopes meet, such as the point *LS* in Figure 3. Thus, the use of this transform makes it far easier to, algorithmically, locate aspects of the waveforms which can be otherwise difficult to find.

The transform, S, is used in two ways. First it is used to locate a point of interest based on the maximum or minimum point of S. This use is demonstrated in Figure 5. Hence, a desired point *P* is defined:

(12) $$P=S_{\text{max}}\;\text{or}\;S_{\text{min}}$$

Both local maximum, $S_{\text{max}(\text{local})}$, and minimum, $S_{\text{min}(\text{local})}$, are also required. $S_{\text{max}(\text{local})}$ exists and is the maximum point of S, if and only if, there exists a maximum stationary point that does not fall at the temporal boundary of S, and similarly for $S_{\text{min}(\text{local})}$.

The second way the shear transform of (6) is used relates to the verification of a particular point given an initial guess. For example, the first guess of the point *MN* is the global minimum of the waveform, after which this point is verified using the shear transformation, resulting in confirmation of the point or a new point to use instead.

This works by locating the maximum of minimum point of the shear transform, *P*_2_, over a given range, *t*, near the first guess, *P*_1_, and also defining a threshold time, *D*. If the point *P*_2_ lies temporally within $t_{P_{1}}\pm D$, then the correct point is the initial guess *P*_1_, otherwise the correct point is *P*_2_.

The choice of $S_{\text{max}}$ or $S_{\text{min}}$, the range of time, *t*, and the threshold time, *D*, are defined for the type of point under consideration. The specific values, listed in Section Point location method, are chosen empirically, based on what features that appear close to the point of interest and the temporal variation that has been observed in these features.

**Figure 6:**
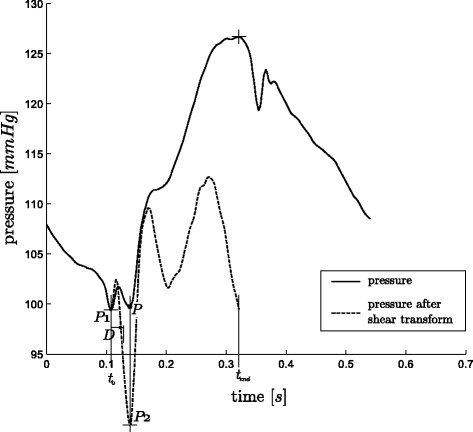
**Use of the shear transform, A.** The desired point for *MN* is *P*. However, in this example the global minimum of the waveform is *P*_1_, which is the initial guess for *MN*. A shear transform of the pressure waveform between *P*_1 _and *MX* reveals a minimum (*P*_2_) outside the range of *D*, and hence the time of *P*_2 _is taken as the time of *MN*.

**Figure 7:**
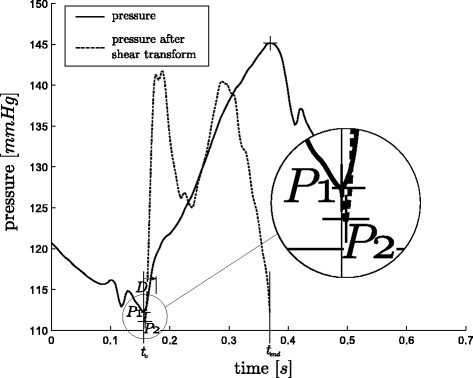
**Use of the shear transform, B.** This example is the other situation in the process of finding *MN* to Figure 6, i.e. the initial guess of the global minimum for *MN* is correct. Here, the minimum of the shear transform from *P*_1 _to *MX* falls within the range of *D * and hence the *P*_1 _is taken as *MN*.

These two situations are graphically shown in Figure 6 and Figure 7 for positive values of *D*. However the same applies for negative values, for which the real point lies before the point *P*_1_, instead of after it. The complete process is defined:

(13) $$\begin{array}{l} P=\left\{\begin{array}{ll} P_{1} & \text{if}\;t_{P_{2}}\;\text{lies temporally within}\;t_{P_{1}}\pm D\\ P_{2} & \text{otherwise} \end{array}\right)\; \end{array}$$

where:

(14) $$\begin{array}{l} P_{1}\equiv \text{initial point to be checked}\\ P_{2}\equiv S_{\text{max}}\;\text{or}\;S_{\text{min}}\;\text{(chosen separately)}\\ \;D\equiv \text{threshold time}\\ \;t\in \{t:t_{0}<t<t_{\text{end}}\} \end{array}$$

Combined these two methods of use, shown in Figure 5 - 7, create a robust and computationally fast method for locating certain hard to find points on a waveform.

### Point location method

**Figure 8:**
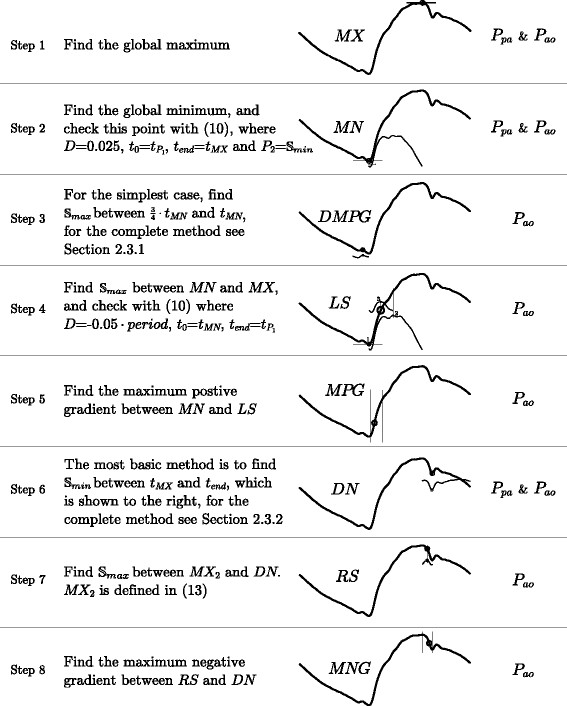
**The method.** The step by step method for finding the points on *P*_*ao *_and *P*_*pa*_, as labelled on the right. The graphics beside each step are for illustration only and are not meant to be part of the definition of the method, rather to see the method in operation on a representative *P*_*ao*_ waveform. Note that the methods described here for *DMPG* and *DN* are note complete as these require a more complex method, refer to Sections Finding DMPG and Finding DN for the complete method for these two points.

The method for finding the points is described in Figure 8 along with the following two sections (Finding DMPG and Finding DN). Figure 8 gives the full text, reproducible and ordered method except for the points *DMPG* and *DN* (which are described in the next two sections) along with a graphical illustration. The graphical illustrations are the out-working of the method for a representative waveform, and are only intended to aid the reader in their understanding of the method, and not to formally describe the method itself. Due to the complexity of the method for the points *DMPG* and *DN*, these two have been described in Figure 8 only for the simplest (as well as the and most common) case, with the full method described in separate sections with relevant figures.

#### Finding DMPG

There are some cases, where *DMPG*, see Figure 3, is equivalent to *MN*. These cases occur when:

(15) $$(t_{\text{MX}}-t_{\text{MN}})<\text{period}·0.25$$

**Figure 9:**
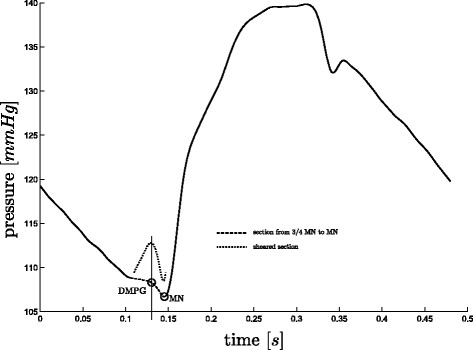
**Finding *DMPG*, A.** A straight forward case for finding *DMPG*, where *P*_1 _of (17) exists, hence *DMPG *≡* P*_1_.

**Figure 10:**
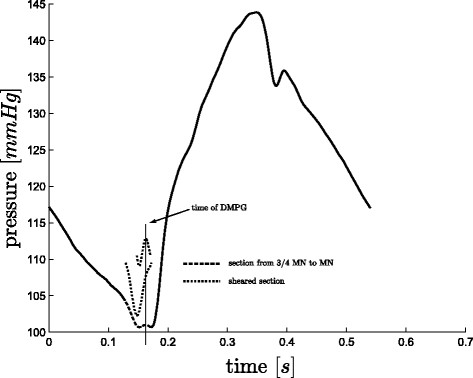
**Finding *DMPG*, B.** A less common case for finding *DMPG*, where *P*_1 _of (17) does not exist, but *P*_3 _does, hence *DMPG *≡* P*_3_.

**Figure 11:**
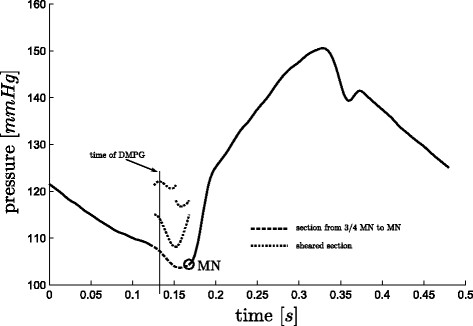
**Finding *DMPG*, C.** A less common case for finding *DMPG*, where *P*_1_, and *P*_3 _of (17) do not exist, but *P*_4 _does, hence *DMPG *≡* P*_4_.

When (15) is not true, *DMPG* is found as the $S_{\text{max}(\text{local})}$ from $\frac{3}{4}·t_{\text{MN}}$ to *MN*, see Figure 9. However, there are a few cases, for both sepsis and pulmonary embolism, in which a local maximum of S does not exist except at the boundaries of the region, which is not acceptable if an automated detection method is desired. In this case, a point *P*_2_, is defined as the $S_{\text{min}}$ from $\frac{3}{4}·t_{\text{MN}}$ to *MN*. If $S_{\text{max}(\text{local})}$ from *P*_2_ to *MN* exists, this is taken as *DMPG*, see Figure 10, otherwise *DMPG* is defined as $S_{\text{max}(\text{local})}$ from $\frac{3}{4}·t_{\text{MN}}$ to *P*_2_, see Figure 11. If this final local maximum does not exist, *DMPG* is defined as $\frac{3}{4}·t_{\text{MN}}$. These cases occur due to noise, variability and dysfunction and are part of what makes robust algorithmic or automated processing difficult.

This process is defined:

(16) $$P=\left\{\begin{array}{ll} P_{1} & \text{if}\;\exists \;P_{1}\\ P_{3} & \text{if}\;\exists \;P_{3}\\ P_{4} & \text{if}\;\exists \;P_{4}\\ \frac{3}{4}·t_{\text{MN}} & \text{otherwise} \end{array}\right)$$

where:

(17) $$\begin{array}{l} P_{1}\equiv S_{\text{max}(\text{local})},\;t\in \{t:\frac{3}{4}·t_{\text{MN}}<t<t_{\text{MN}}\}\\ P_{2}\equiv S_{\text{min}(\text{local})},\;t\in \{t:\frac{3}{4}·t_{\text{MN}}<t<t_{\text{MN}}\}\\ P_{3}\equiv S_{\text{max}(\text{local})},\;t\in \{t:t_{P_{2}}<t<t_{\text{MN}}\}\\ P_{4}\equiv S_{\text{max}(\text{local})},\;t\in \{t:\frac{3}{4}·t_{\text{MN}}<t<t_{P_{2}}\} \end{array}$$

#### Finding DN

**Figure 12:**
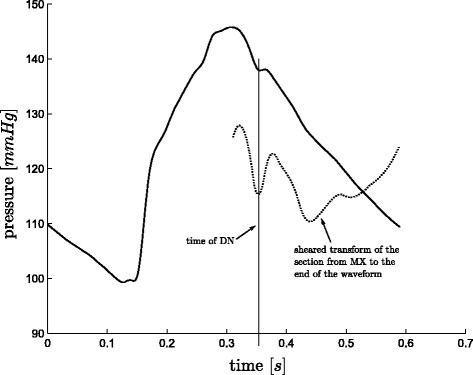
**Finding *DN*.** An example of where the first local minimum of the shear transform is the correct time for the point *DN*.

The general approach to find the point *DN*, see Figure 3, is to find $S_{\text{min}}$ between *t*_*MX*_ and *t*_*end*_ (or *period*). However, in a number of cases this approach fails due to oscillations towards the end of the waveform, see Figure 12. Also, using only the first local minimum (as is the case in Figure 12) works only in a few cases and therefore is not a robust solution either. Hence a more specific algorithm is required.

A second maximum point is defined:

(18) $$\begin{array}{l} MX_{2}\equiv \text{max point of}\;P_{\mathrm{ao}}(t),\\ \;\;t\in \left\{t:t_{\text{MN}}+\frac{\text{period}}{5}<t<\text{period}\right\} \end{array}$$

and two more intermediate points are defined:

(19) $$DN_{1}\equiv \text{lowest}\;S_{\text{min}(\text{local})},t\in \{t:t_{MX_{2}}<t<\text{period}\}$$

(20) $$DN_{2}\equiv \text{first}\;S_{\text{min}(\text{local})},t\in \{t:t_{MX_{2}}<t<\text{period}\}\;$$

From *DN*_1_ and *DN*_2_, the real *DN* is chosen, defined:

(21) $$\text{DN}=\left\{\begin{array}{ll} DN_{1} & \text{if}\;(C_{A}\;\wedge \;C_{B})\;\vee \;(C_{C}\;\wedge \;C_{D})\\ DN_{2} & \text{otherwise} \end{array}\right)$$

where:

(22) $$\begin{array}{l} C_{A}\equiv DN_{2}>MX_{2}\\ C_{B}\equiv (t_{DN_{2}}-t_{MX_{2}})<0.15·\text{period}\\ C_{C}\equiv \frac{DN_{2}-DN_{1}}{P_{\mathrm{ao}}(t_{\text{MX}})}>0.02\\ C_{D}\equiv (t_{DN_{2}}-t_{MX_{2}})<0.11·\text{period} \end{array}$$

While using a measured *P*_*pa*_ waveform, *DN* is defined:

(23) $$\text{DN}=DN_{1}$$

#### Validation Test

The method presented was developed on a set of five pigs (51 waveforms) that were induced with pulmonary embolism [32,33], and then independently tested on a further five pigs (37 waveforms) induced with septic shock, and treated with haemofiltration [34,35].

The points for all waveforms (see Figures 3- 4) were identified or checked individually by eye. The two gradients (*MPG* and *MNG*) were first located through simple computation, and then individually checked by eye and corrected were necessary. Due to the nature and location of these points (maximum gradient of a sigmoidal function), they are the two easiest and most reliable to find algorithmically, and in fact the algorithmic approach is more accurate than hand selection. The two shoulders (*LS* and *RS*) were first located through the algorithm developed prior to that which is described in this paper, after which each point was individually checked and corrected. Because there is no formal definition for the location of these “shoulder” points, it was left to an algorithmic definition. For a validation test this definition is self fulfilling. However, as *LS* (*RS* is only used to aid in finding *MNG* , and is hence not included in the validation results) is found as an intermediate step to the estimation of the cardiac elastance, its full and more formal validation would be the results of the cardiac estimation which is not in the scope of this paper. All the remaining points were hand selected.

The automated method was applied to the waveforms and the identified points assessed against the known points for accuracy in time. The use of separate data with different cardiac dysfunction to design and test the method ensures the robustness of the validated method.

## Results

**Figure 13:**
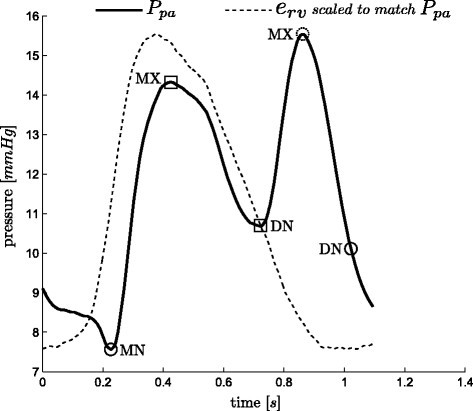
**Where the method fails.***P*_*pa*_ alongside the matching TVE. The automatic or algorithmic method failed to capture the correct *DN* point (circle), the real *DN* and associated *MX* are marked by squares.

For the points (*MN* and *DN*) required when using *P*_*pa*_ (Figure 4), the method located both points in 87 of the 88 waveforms to within the sample frequency of 200Hz (0.005 sec), missing *DN*, from one waveform. This missed point is in a waveform at the start of the third pig of the sepsis cohort and is unique to the data set, both in the measured TVE and *P*_*pa*_, as shown in Figure 13, compared to the more typical *P*_*pa*_ waveform in Figure 4. The failure is due to the unusual second peak of *P*_*pa*_, and the early decay of the TVE.

For each *P*_*ao*_ waveform, the method locates eight points, *MX*, *MN*, *DMPG*, *LS*, *MPG*, *DN*, *RS*, *MNG*. However, *RS* and *MN* are only used to aid the location of other points. These two points were both located sufficiently to enable the method to progress in all 88 waveforms. Results for the other six points are shown in Table 1. Of 616 total points, 605 were found within 1%, 5 within 5%, 4 within 10% and 2 within 20%.

**Table 1:** The error data for each location, grouped by number of points found per error band for each location

	**<1*%***	**1*−*5*%***	**5*−*10*%***	**10*−*20*%***
*DMPG*	84	1	3	0
*MPG*	88	0	0	0
*LS*^∗^	86	1	1	0
*MX*	88	0	0	0
*MNG*	87	0	0	1
*DN*	86	2	0	0
TOTAL	605	5	4	2

Accuracy of the method: number of points grouped by absolute error (of 88 total points). ^*^ note that the validation *LS* here is partially self fulfilling and only included here for completeness.

## Discussion

The automated, algorithmic method presented enables the mapping between aortic pressure (*P*_*ao*_), pulmonary artery pressure (*P*_*pa*_), and the ventricle TVE (*e*_*rv*_(*t*) and *e*_*lv*_(*t*)), by accurately processing the *P*_*ao*_ and *P*_*pa*_ waveforms to identify specific points. Once combined, they enable a very useful tool, for clinicians to obtain very accurate TVE without further invasive or risky sensors or procedures.

There are other ways to locate the points on the pressure waveforms, most notably a derivative and second derivative method. However, this becomes problematic in practice due the noise inherent in the waveforms. The method that has been developed in this paper, was designed to work with the level of noise that is typically seen on these measurements, and is therefore more involved than a simple derivative method.

The method presented was robust to the typical and significant variation and noise in *P*_*ao*_ and *P*_*pa*_ waveforms. The method was developed on five pigs induced with pulmonary embolism, and then tested independently with data from another set of five pigs induced with septic shock. The results give confidence that this method will generalize to a wider set of disease states and to human data.

However, while the results were very good, this research needs further validation on a wider cohort of pigs and types of dysfunction to further quantify the limits and accuracy of this approach. Direct validation on humans is the ultimate goal. However, the results appear robust, and justify and enable a wide range of further more in-depth validation studies of both the method and its potential uses when reconstructing TVE for monitoring and diagnosis.

Clinically, it must be noted that for this method to work a Swan-Ganz catheter is assumed. If radial artery pressure was measured instead, there would be more oscillations in the waveform, potentially requiring modifications. However, Swan-Ganz catheters are still commonly used, and this application would add value to their use, which is otherwise sometimes contested [6-8].

The method developed in this paper shows promise for gaining clinical insight and improving diagnosis. It can enable clinicians to get more information about the current patient state, without the use of more invasive measurements, as well as beat-to-beat tracking of this information. This level of detail is far more than currently available and could potentially lead to better and earlier diagnosis of dysfunction, as well as better knowledge of response to treatment, non-invasively, as it needs no further procedures or sensors required.

## Conclusions

This paper has presented a robust, potentially dysfunction-independent method to find the waveform points necessary to use proven methods to non-invasively and automatically estimate the otherwise unavailable left and right ventricle TVEs with accuracy well within measurement error. This capability is enabled using standard measurements that are already commonly used in an intensive care setting, thus involving no additional risk to the patient. The results thus justify prospective validation of these conclusions.

## Competing interests

The authors declare that they have no competing interests.

## Author’s contributions

DS drafted the manuscript and developed the algorithm. JR and JC participated in the algorithm development with added input from TD. CH participated in the initial mathematical formulation. GS provided physiological understanding and clinical input at all stages. BL, AG, PK and TD provided the porcine data and further clinical input and relevance. JC and TD edited and aided the writing of the manuscript and revisions with DS. All authors read and approved the final manuscript.
